# Community Engagement to Optimize the Use of Web-Based and Wearable Technology in a Cardiovascular Health and Needs Assessment Study: A Mixed Methods Approach

**DOI:** 10.2196/mhealth.4489

**Published:** 2016-04-25

**Authors:** Leah R Yingling, Alyssa T Brooks, Gwenyth R Wallen, Marlene Peters-Lawrence, Michael McClurkin, Rebecca Cooper-McCann, Kenneth L Wiley Jr, Valerie Mitchell, Johnetta N Saygbe, Twanda D Johnson, Rev. Kendrick E Curry, Allan A Johnson, Avis P Graham, Lennox A Graham, Tiffany M Powell-Wiley

**Affiliations:** ^1^ Cardiovascular and Pulmonary Branch Division of Intramural Research, National Heart, Lung, and Blood Institute National Institutes of Health Bethesda, MD United States; ^2^ Clinical Center National Institutes of Health Bethesda, MD United States; ^3^ Office of the Clinical Director Division of Intramural Research, National Heart, Lung, and Blood Institute National Institutes of Health Bethesda, MD United States; ^4^ Division of Genomic Medicine National Human Genome Research Institute National Institutes of Health Bethesda, MD United States; ^5^ Pennsylvania Avenue Baptist Church Washington, DC United States; ^6^ College of Nursing and Allied Health Sciences Howard University Washington, DC United States

**Keywords:** mHealth, physical activity, community-based participatory research, obesity, African Americans, activity monitoring, qualitative research, focus groups, community

## Abstract

**Background:**

Resource-limited communities in Washington, D.C. have high rates of obesity-related cardiovascular disease in addition to inadequate physical activity (PA) facilities and limited Internet access. Engaging community members in the design and implementation of studies to address these health disparities is essential to the success of community-based PA interventions.

**Objective:**

The objective of the study was to use qualitative and quantitative methods to evaluate the feasibility and acceptability of PA-monitoring wristbands and Web-based technology by predominantly African American, church-based populations in resource-limited Washington, D.C. neighborhoods.

**Methods:**

To address cardiovascular health in at-risk populations in Washington, D.C., we joined community leaders to establish a community advisory board, the D.C. Cardiovascular Health and Obesity Collaborative (D.C. CHOC). As their first initiative, the Washington, D.C. Cardiovascular Health and Needs Assessment intends to evaluate cardiovascular health, social determinants of health, and PA-monitoring technologies. At the recommendation of D.C. CHOC members, we conducted a focus group and piloted the proposed PA-monitoring system with community members representing churches that would be targeted by the Cardiovascular Health and Needs Assessment. Participants (n=8) agreed to wear a PA-monitoring wristband for two weeks and to log cardiovascular health factors on a secure Internet account. Wristbands collected accelerometer-based data that participants uploaded to a wireless hub at their church. Participants agreed to return after two weeks to participate in a moderated focus group to share experiences using this technology. Feasibility was measured by Internet account usage, wristband utilization, and objective PA data. Acceptability was evaluated through thematic analysis of verbatim focus group transcripts.

**Results:**

Study participants (5 males, 3 females) were African American and age 28-70 years. Participant wristbands recorded data on 10.1±1.6 days. Two participants logged cardiovascular health factors on the website. Focus group transcripts revealed that participants felt positively about incorporating the device into their church-based populations, given improvements were made to device training, hub accessibility, and device feedback.

**Conclusions:**

PA-monitoring wristbands for objectively measuring PA appear to be a feasible and acceptable technology in Washington, D.C., resource-limited communities. User preferences include immediate device feedback, hands-on device training, explicit instructions, improved central hub accessibility, and designation of a church member as a trained point-of-contact. When implementing technology-based interventions in resource-limited communities, engaging the targeted community may aid in early identification of issues, suggestions, and preferences.

**ClinicalTrial:**

Trial Registration: ClinicalTrials.gov NCT01927783; https://clinicaltrials.gov/ct2/show/NCT01927783 (Archived by WebCite at http://www.webcitation.org/6f8wL117u)

## Introduction

### Cardiovascular Disease in the United States

Cardiovascular disease (CVD) is the leading cause of death in the United States [1]. It is recognized that modifiable lifestyle risk factors, including insufficient physical activity (PA), are associated with increased risk of adverse health outcomes from CVD [2,3]. Despite the considerable health benefits associated with regular participation in PA, less than 25% of US adults meet the prescribed PA guidelines of at least 30 minutes of moderate-intensity aerobic activity five days per week, 20 minutes of vigorous-intensity aerobic activity three days per week, or an equivalent combination of the two, plus muscle-strengthening activities on at least two days per week [4]. Of particular concern are individuals in economically disadvantaged and resource-limited communities, who are more likely to report being physically inactive and suffer disproportionately from obesity and obesity-related cardiovascular risk factors [5-7].

### Physical Activity Interventions

Recognizing the potential for broad impact and sustainability, recent work has promoted population-based strategies for improving PA levels [8]. Community-based programs may be an effective population-based strategy for delivering PA interventions in underserved, economically disadvantaged communities, as they have demonstrably leveraged the local built environment and empowered community members [9]. Methods for implementing a PA intervention in a community-based setting vary across studies. In a review of existing community-based interventions promoting PA and healthy eating, all studies targeting adult populations implemented a PA component [10]. Of those PA interventions set in specifically resource-limited communities, most relied on self-report and did not include objective PA measures (eg, accelerometers, pedometers) [10]. Of the reviewed interventions set in specifically resource-limited communities, none used emerging health technologies that provide feedback, such as wrist-worn electronic activity monitors to objectively measure PA.

Wearable, electronic activity monitors have been identified recently as a potential tool to integrate into population-based PA interventions [11,12]. Electronic activity monitor systems have been defined previously as a wearable device that objectively measures lifestyle PA and can provide feedback, beyond the display of basic activity count information, via the monitor display or through a partnering application to elicit continual self-monitoring of activity behavior [13]. They offer the potential to extend PA interventions beyond the clinical setting to those with limited access to care; however, the feasibility of incorporating electronic activity monitors and Web-based technology as part of a PA intervention in community-based settings is unknown. Thus, evidence-based PA interventions that use technology in resource-limited, community-based programs warrant investigation. Understanding how electronic PA monitors can be optimized in community-based interventions may reveal opportunities to increase PA, to reduce cardiovascular (CV) health disparities, and to improve clinical outcomes among economically disadvantaged populations.

Community leaders in our target population proposed the engagement of church members to pilot test the feasibility and acceptability of using technology to evaluate health behaviors in the resource-limited Washington, D.C., communities. Therefore, we conducted a mixed methods pilot study to: (1) evaluate the use of an electronic PA-monitoring wristband for objectively measuring PA and the use of Web-based technology for monitoring CV health factors; (2) illuminate advantages and disadvantages of implementing technology-based PA interventions in a resource-limited setting; and (3) explore how community-based participatory research (CBPR) can shape PA interventions in resource-limited, community-based programs.

## Methods

### Study Approval

The NHLBI Institutional Review Board (National Institutes of Health, NIH, Protocol 13-H-0183) approved the CV Health and Needs Assessment and the CV Health and Needs Assessment Qualitative Study. All participants provided written informed consent.

### Study Design

**Figure 1:**
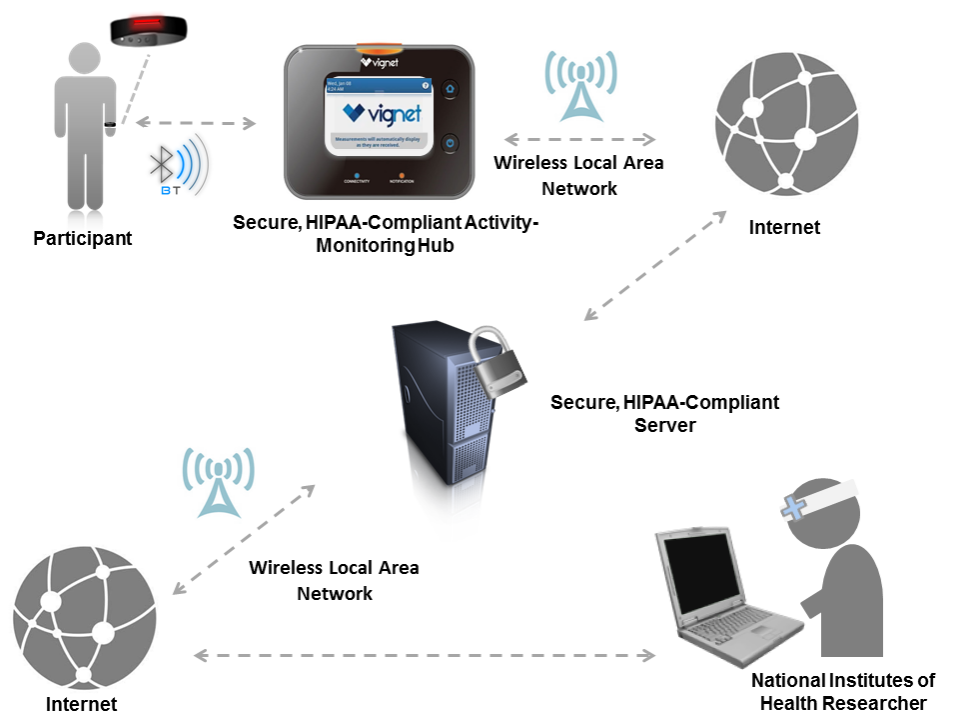
Secure data collection process; Cardiovascular Health and Needs Assessment Qualitative Study, 2014. HIPAA: Heath Insurance Portability and Accountability Act.

We conducted a CBPR mixed methods study that incorporated a moderated focus group and pilot testing of a two-part PA-monitoring system: a PA-monitoring wristband (Dynamo Activity Tracker, Oregon Scientific, Tualatin, OR) with a centralized hub for data download in a community location, and a secure Internet account for manual tracking of CV health factors (Vignet Corp, McLean, VA). Figure 1 shows the data collection process. The particular PA-monitoring system featuring a Health Insurance Portability and Accountability Act-compliant centralized hub was selected to address secure data transfer issues and potential technology access barriers. The selected system made secure uploading and viewing data possible for all participants regardless of computer, mobile device, or Internet access.

To consult on the planning and implementation of a community-based initiative, we established the D.C. CV Health and Obesity Collaborative (D.C. CHOC), a community advisory board (CAB) comprised of a diverse group of community leaders, church leaders, and coinvestigators. It was at the recommendation of members of the D.C. CHOC that we conducted a focus group and pilot testing with a sample from the target church-based population for feedback on the proposed PA monitor, prior to testing on a larger population in the CV Health and Needs Assessment. The pilot testing that is the focus of this study was called the CV Health and Needs Assessment Qualitative Study.

The focus group was conducted after two weeks of PA monitoring, a testing period commensurate with similar mobile and mobile phone-based activity tracking studies [14,15]. The moderator(s) sought insight into participants’ experiences using the wrist-worn PA monitor, the hub for PA monitor data upload, and the Web-based account for monitoring PA and other CV health factors. The outcomes of interest in our study were: (1) feasibility of the PA monitoring system as measured by Internet account input, wristband utilization frequency, and objective PA data and (2) acceptability of the system as measured by results of a moderated focus group discussion designed to elicit participants’ opinions about their experiences with the device and to prompt their suggestions for incorporating similar technologies in future behavioral weight loss interventions within their communities.

### Study Population

Participants were recruited from December 2013 to January 2014 from three churches in Wards 5, 7, and 8 of Washington, D.C. These are three Washington, D.C. wards with a median household income significantly lower than all of Washington, D.C. and where resources for PA and healthy nutritional options are most limited [16]. Participants were congregants at one of the participating churches, age 19-85 years, provided informed consent, and possessed sufficient English language proficiency to carry out study tasks. No more than 15 participants were recruited for the study, and 7-9 participants were anticipated, a total that is within the recommended range for qualitative research group discussion and is comparable to other mobile health (mHealth) and electronic health (eHealth) initial pilot testing groups [14-18].

### Physical Activity Monitoring System

#### Physical Activity Monitoring Wristband

**Figure 2:**
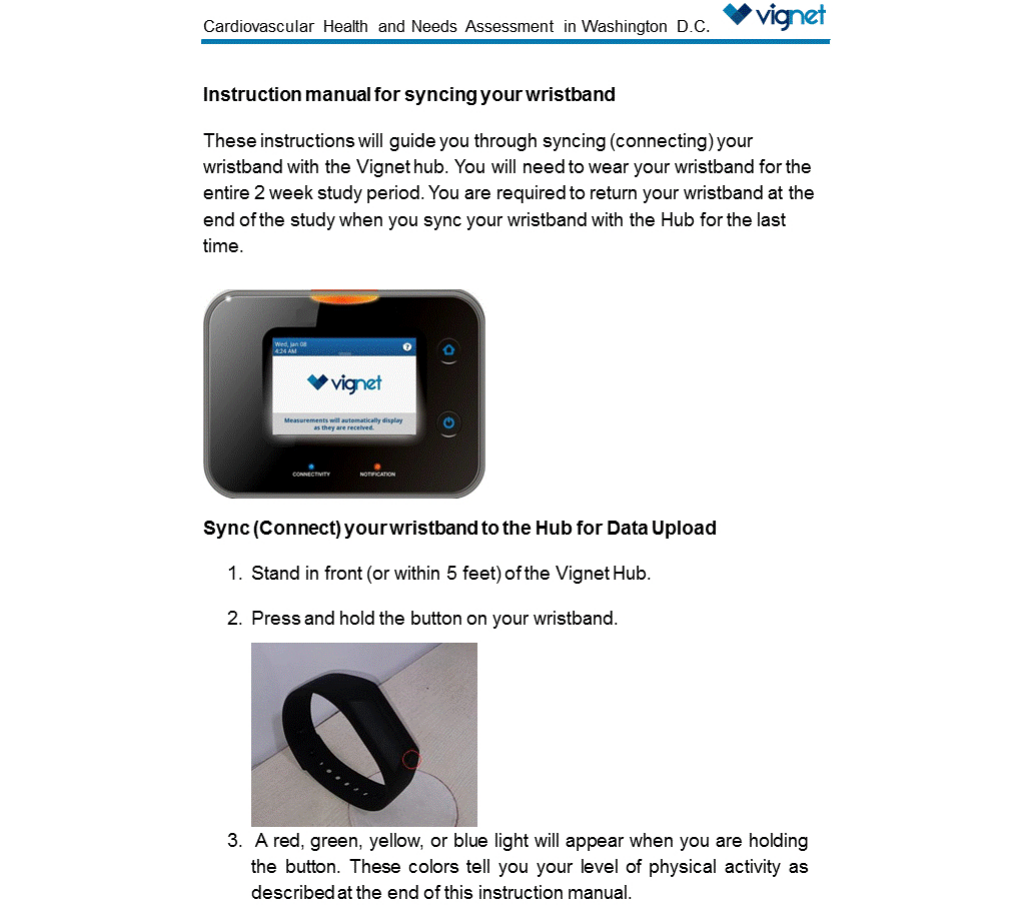
Instruction manual (page 1) for syncing physical activity-monitoring wristband with hub; Cardiovascular Health and Needs Assessment Qualitative Study, 2014.

Each participant was provided a wrist-worn wireless activity and sleep wristband to collect and self-monitor PA and sleep duration for two weeks. A thirty-minute wristband training session was provided on the day of device distribution, and a written instruction manual (Figure 2 shows this) was distributed to all participants.

**Figure 3:**
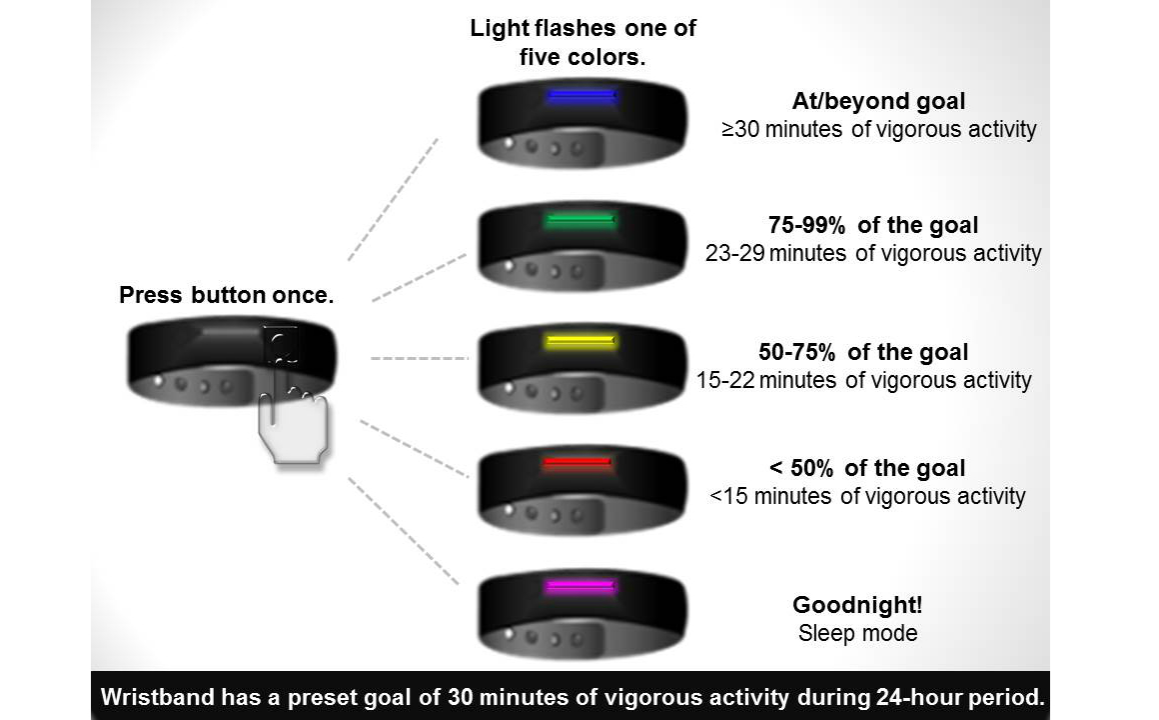
Physical activity-monitoring wristband colored light system; Cardiovascular Health and Needs Assessment Qualitative Study, 2014.

The wrist-worn PA monitor collected accelerometer-based data on the amount and intensity of PA (eg, steps taken, calories burned, distance travelled, and minutes of vigorous activity). The wristband used a colored-light system (Figure 3 shows this) to communicate PA progress to the participant. Though participants were instructed not to modify their routine PA, the wristband featured a preset goal of 30 minutes of vigorous activity throughout a 24-hour period. Pressing the wristband button prompted the wristband light to display a specific color. The various colors indicated sleep mode, battery depletion, or progress toward PA goals.

#### Physical Activity Monitoring Data Collection

**Figure 4:**
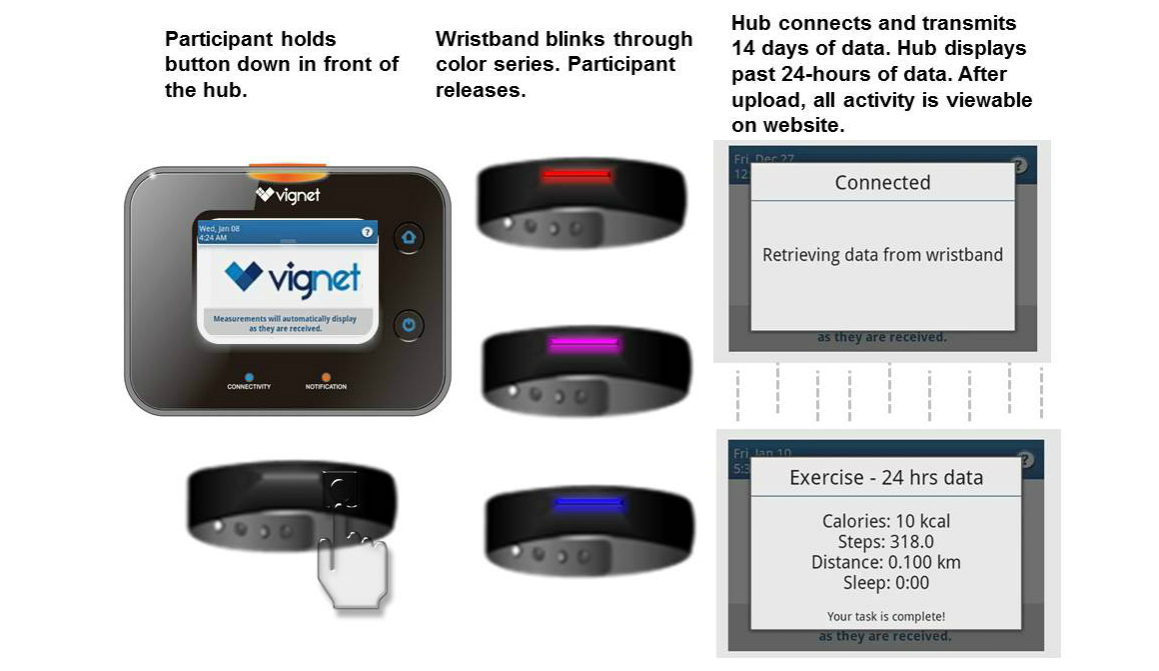
Physical activity-monitoring wristband with hub; Cardiovascular Health and Needs Assessment Qualitative Study, 2014.

All participants uploaded recorded PA and sleep data wirelessly from their wristband devices to the centralized hub on the final day of the study. The hub captured and transmitted the previous fourteen days of PA data. After a successful upload, the hub displayed the past 24-hours of PA data to the participant. Participants were able to synchronize their wristbands with the hub at any point during the study period. After successfully synchronizing their wristbands with the hub, participants had access to their recorded PA accelerometer-based data in addition to all self-logged data on a website. Figure 4 shows the wristband synchronizing process. A hub was provided to each church.

#### Internet Account for Tracking Physical Activity Data

Each participant was provided with a secure account (using a deidentified username and password) on a website associated with the PA-monitoring wristband. Participants were trained on general website use and how to log self-reported PA, weight, dietary intake, heart rate, blood pressure, and blood glucose levels, if measured. Self-reporting of these measures was optional, and participants could do so from a personal or church-based computer. Investigators monitored usage of the wristband and website by collecting deidentified data from a website during the two-week study.

### Focus Group

At the end of the two-week period, participants participated in a moderated focus group to provide feedback on their experiences using the PA-monitoring wristband, the hub, and the Internet account. Participants were remunerated with a US $25 gift card, compatible with time required for the focus group.

One moderator, who acted as a facilitator, led the focus group. There were two comoderators that assisted with the focus group. The moderator led the discussion using a Moderator’s Guide (see Multimedia Appendix 1), which included preselected questions and probes. The questions were grouped in categories recommended by Krueger [19]: opening, introductory, transition, key, and closing. The comoderators recorded notes, made observations, and managed the equipment (tape recorder, microphones, etc).

### Quantitative Data Analysis

Quantitative accelerometer-based data were collected from all participants’ wristbands on the final day of the study. All quantitative analyses were performed in SAS version 9.3 (SAS Institute Inc, Cary, NC, USA).

### Wristband and Website Utilization Frequency

Wristband utilization frequency was measured by the number of days with wristband-measured activity. The number of self-logged entries per participant measured Internet account usage.

### Objective Measurements

Quantitative data included steps taken (measured in strides), distance travelled (measured in miles), vigorous activity (measured in minutes), calories burned (measured in kilocalories), and sleep time (measured in hours). The final day of collected data was omitted from the analysis, as it represented only a partial day and likely was not representative of a typical full day’s PA measurements. Days with no recorded PA data were considered “missing” and were not included when calculating average PA measures per day.

### Qualitative Data Analysis

The focus group was audio-recorded, and the recording was transcribed verbatim by an independent clinical research organization (Social Solutions International, Inc, Silver Spring, MD, USA). A member of the research team, who listened to the audio files to verify they were transcribed verbatim, performed an internal reliability check on the transcript. As a preliminary step in the qualitative thematic analysis, four members of the research team developed a codebook, or dictionary of themes, based on participant responses. Four coders, who independently reviewed the interview transcripts, assessed evidence of each code or theme. Each coded theme was accompanied by an operational definition that allowed for clarity and consistency in the coding process. After data were transcribed and cross-checked by the four coders, NVivo (version 9.0) was utilized for further qualitative analysis. Discordant coding was discussed until consensus among the four coders was achieved. Once the iterative process of consensus building was complete, an NIH intramural qualitative research expert validated the themes and coding.

To ensure that the trustworthiness of the qualitative data was preserved, three criteria were used to assess rigor: “creditability”, “auditability”, and “fittingness” [20], as shown in Multimedia Appendix 2 (see Multimedia Appendix 2). An intramural mixed methods expert, to ensure creditability, validated the themes (ie, truth of the findings) [20]. To maintain auditability (ie, “the adequacy of the information leading the reader from the research question and raw data through various steps of analysis to the interpretation of findings”) [20] and fittingness (ie, “faithfulness to everyday reality of participants”) [20] of the qualitative data, a thorough description of the interview setting was reported and selected quotes that are illustrative of each designated theme are displayed in the tables to highlight pertinent findings.

## Results

### Demographic Characteristics

There were eight individuals that participated in the two-week pilot testing period and the subsequent focus group. Among the participants, 63% (5/8) were male, the mean age was 53.3 ±12.2 years, all participants were African American, and all attained higher than a high school education. Demographic characteristics for the study population are presented in Table 1.

**Table 1:** Participant baseline characteristics in the CV Health and Needs Assessment Qualitative Study, 2014 (n=8).

Variable		
Sex, n (%)		
	Female	3/8 (37)
	Male	5/8 (62)
Age, years			
	Mean (SD)	53.3 (12.2)
	Range, years	28-70
Race, n (%)			
	Black/African American	8/8 (100)
Marital status, n (%)			
	Single	1/8 (12)
	Married	7/8 (87)
Education, n (%)		
	Some college	3/8 (37)
	College degree	2/8 (25)
	Technical degree	1/8 (12)
	Graduate/professional degree	2/8 (25)
Annual household income US (n=7), n (%)		
	< $60,000	2/7 (28)
	≥ $60,000	5/7 (71)

### Quantitative Data

#### Wristband and Website Utilization Frequency

Of the 13 possible full days of PA-monitoring wristband readings, participant wristbands provided readings for 10.1 ± 1.6 days. Most participants (63%, n=5/8) registered 10 days or more of PA data. There were five participants (63%, n=5/8) that utilized the wristband’s sleep function more than once. Due to a system issue, depleted battery, or lack of participant compliance, only 12% (n=1/8) of participants had a complete 13-day dataset. There were two participants (25%, n=2/8) that used the website to track additional CV-related health factors by logging body weight, blood glucose level, PA minutes, and hours of sleep, or hours of sleep.

#### Objective Measurements

**Figure 5:**
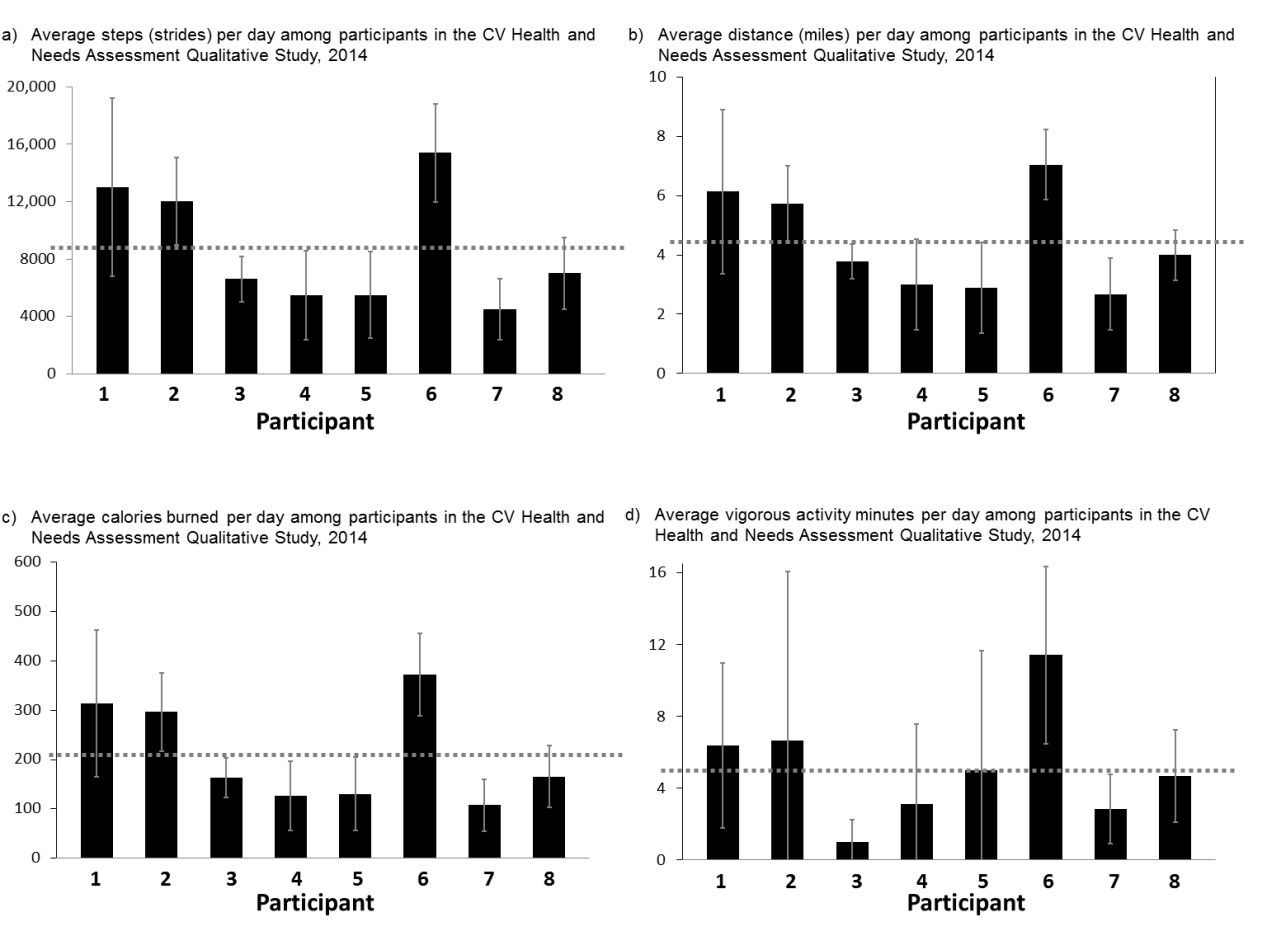
Quantitative participant (n=8) wristband data — Cardiovascular Health and Needs Assessment Qualitative Study, 2014. M=Mean; a) Overall average steps, M(SD) = 8693 (3124) strides per day; b) Overall average distance, M(SD) = 4.40(1.36) miles per day; c) Overall average calories burned, M(SD) = 210(76.6) calories per day; and d) Overall average vigorous activity minutes, M(SD) = 5.13(4.47) minutes per day.

Figure 5 shows average daily steps, distance, calories burned, and vigorous minutes for each participant. For the days on which steps were registered, mean steps per day among participants was 8693 ± 3124 steps. Among participants, the maximum steps per day were 15,417 ± 3420 steps and the minimum was 4155 ± 2323 steps. For the days on which data were measured, mean distance travelled per day among participants was 4.40 ± 1.36 miles, mean calories burned per day was 210 ± 76.6 calories, and mean vigorous activity minutes per day among participants was 5.13 ± 4.47 minutes. There were six participants (75%, n=6/8) that registered at least one day with no vigorous activity minutes.

### Qualitative Data

There were eight themes that emerged from the focus group and are shown in Textbox 1. Selected quotes from participants associated with each theme and subtheme are presented in Table 2.

**Focus group themes and subthemes - CV Health and Needs Assessment Qualitative Study, 2014.**

- Feedback
  Desire for immediate device feedback
  Accessibility of feedback about PA
- Design of PA Monitor
  Physical discomfort
  Features
- Ambiguity over project goals
- Issues about hub
- Website for supporting PA monitors
- Feasibility of using PA-monitoring system
- Suggestions for improvement

**Table 2:** Focus group themes, subthemes, and quotes - CV Health and Needs Assessment Qualitative Study, 2014.

Theme and subthemes		Illustrative quotes
Feedback		
	Desire for immediate device feedback	That’s the fundamental problem...there’s no immediate feedback...We need some kind of feedback besides these lights. [Male, age 49] I was thinking about what [participant name] said about the vibration...Maybe something to vibrate when I’m at 50, 60 or 100% [of daily recommended physical activity]. [Male, age 55]
	Accessibility of feedback about PA	And I’m not always near a hub so the only feedback I had were those lights. [Male, age 49] The definition of what’s vigorous...What does that really mean? I work out 45 minutes every day. I lift, do treadmill and do crunches but my arm’s not moving enough for you. [Male, age 55]
Design of PA monitor		
	Physical discomfort	I had no way to clip this device like I clip on my watch...During the day I always had to touch it to make sure it didn’t come off. Maybe a different kind of clamp with like a watch clamp. [Male, age 55] I had major skin irritations from it. From the metal part that touched my arm. I still have irritation now. [Female, age 59]
	Features	Even though it says purple sometimes it looked more like red to me, so I don’t know...The colors should be more distinct. [Male, age 49] The device was kind of bulky around my wrists. I was trying to put my shirt on, move the device up and down my wrist to make sure I could get my shirt over it or keep my shirt over top of it. [Male, age 55]
Ambiguity over project goals		What I understood...is that you measure what you do normally; don’t do anything extra or less. [Female, age 62] The goal of this was to show the green. That’s why I thought we were working so hard to get it done. [Male, age 55]
Recording PA		We just took the band off to sleep because...the light was a problem the first night...When I went on the computer, I put how many hours I slept. [Female, age 62] A lot of my activity’s in the water, and since we couldn’t do that you have to put them in the computer. [Female, age 62]
Issues about hub		[Our hub] didn’t work at our church at all. [Female, age 69] We had to go outside to one of our other buildings to try and sync it...The timing trying to get it just right between the two services [was challenging]. [Male, age 59]
Website for supporting PA monitors		I couldn’t pull it up on my phone, which is where I am most of the time. [Female, age 43] I would have preferred to have an app. [Male, age 49]
Feasibility of using PA-monitoring system		I think a centrally located area in church that’s not lock-and-key will make the user...more like, “Okay, I can sit there and download it”, not have to run around, “Who has the key?”, they stand there while you’re doing it, then they lock up the room when you leave. [Male, age 55] I was as at church but not able to get to the actual [hub], because where I was located at that point in time. Because...you know I have a lot going on. So it’s like I can get it this point in time or just can’t. [Female, age 62]
Suggestions for improvement		You may want...a point person in the church that has been trained in the docking system, so they don’t have to call you all the time, train somebody in the church so they can troubleshoot for you real quick. [Male, age 55] Maybe social media...so we can share our results. [Male, age 49]

### Focus Group Themes

Regarding the feedback theme, multiple participants reported a desire for immediate, more accessible feedback complementary to the colored lights on the wristband. Additionally, participants tended to be disappointed with their personal vigorous activity minutes and requested more information on the types of activity that would register as vigorous.

Regarding the PA monitor design; participants raised concerns about physical discomfort and the monitor features. Some participants recommended a different clasp to prevent accidental detachment, and two participants experienced skin irritation where the wristband was secured. Several participants experienced difficulty differentiating between the colored lights, and a few participants found the wristband to be bulky in size.

An overarching theme was the ambiguity of the project goals outlined in the initial training. During training, participants were instructed not to modify their routine PA; however, the preset 30-minute vigorous activity goal of the wristband led some participants to modify their activities in pursuit of “show[ing] green” by reaching 30 minutes of vigorous activity in a single 24-hour period. In relation to the theme of recording PA, those participants who engaged in activities not measured by the wristband (eg, water sports, weight lifting, etc) preferred if the wristband would have captured all PA, regardless of its nature.

Participants also discussed issues about the centralized hub and its availability for synchronizing. There was one participant that reported that the hub did not work at his church, which restricted synchronizing during the two-week period. At another church, the hub was reported to be inconvenient to access.

Regarding the website for supporting the PA monitor, most participants reported no use of the site and preferred alternative options for manual entries and self-monitoring, including a mobile phone application (app) for tracking CV health factors and PA. With regards to implementation of the PA-monitoring system, participants suggested identifying a trained point-person for each church, and sharing PA data through social media to add a competitive element and an aspect of support. Additionally, participants reported that the feasibility of the PA-monitoring system would be contingent on the implementation of specific changes, such as moving the hub to a central area, extending hub hours, and identifying a point-person within the church for expedited troubleshooting.

### Changes Made

The changes made for future testing of the PA technology in Washington, D.C., community-based populations based on the qualitative focus group data are documented in Table 3. We addressed several concerns by modifying how the PA wristband monitor training was conducted in the larger Washington, D.C., CV Health and Needs Assessment. We also revised the information provided during the PA wristband monitor training and in the wristband instruction manual. Addressing the ambiguity of “vigorous activity”, we provided a clearer and more relatable definition of vigorous activity to participants (ie, activities that require hard physical effort and cause large increases in breathing or heart rate such as running; aerobics; using the elliptical machine, with arms; or playing a sport like football, basketball, soccer, or tennis). To address design concerns of the PA monitor, we ensured proper wristband attachment during training and warned participants of potential skin irritation issues. Participants were advised to wear the wristband loosely. To address potential misunderstandings of the colored-light system, we incorporated a detailed description of the wristband’s colored lights during device training and on the “Helpful Hints” sheet, a frequently asked questions sheet created for at-home reference.

**Table 3:** Lessons learned and changes made for testing PA technology in broader community-based populations.

Theme and subthemes		Changes made
Feedback		
	Desire for immediate device feedback	Taught participants how to use the wristband’s colored lights to monitor activity minutes (ie, lights progress through series of colors as participant approaches 30 minutes of vigorous activity) *Red*=less than 50% of goal *Yellow*= 50-75% of goal *Green*=75-99% of goal *Blue*=100% or above goal
	Accessibility of feedback about PA	Provided clear definition of vigorous activity in both the training and instruction manual (ie, activities that require hard physical effort and cause large increases in breathing or heart rate, eg, running, aerobics, using the elliptical machine, with arms, or playing a sport like football, basketball, soccer, or tennis)
Design of PA monitor		
	Physical discomfort	Informed participants about wristband-related skin irritation during device training and in the instruction manual Instructed participants to wear the wristband loosely if irritation is likely Ensured that participants latched wristband properly during device training
	Features	Incorporated detailed section on the wristband’s colored lights during device training and on the “Helpful Hints” for at-home reference
Ambiguity over project goals		Redesigned education component to explicitly state project goals (ie, participants should continue with routine PA and not change behavior) Explicitly stated project goals in written instruction materials Developed two instructional training videos on device and hub usage that were used during device training and made publically available after the event for participant reference Incorporated more hands-on in-person training where participants could use the website, test wristband lights, and upload wristband data
Recording PA		Instructed participants to test sleep mode during device training Educated participants on the use of sleep mode, but purposefully did not emphasize its use Tested website to manually input sleep and PA during device training Developed instructional video on recording PA that was used during hub and device training and was made publically available during the study for participant reference
Issues about hub		Corresponded weekly with device company and participants to identify and troubleshoot hub issues Incorporated troubleshooting report sheet next to hub for streamlined reporting Provided participants with a schedule of “hub hours” and the option to synchronize wristbands at any of the participating churches Identified a point-person within church community to aid in troubleshooting minor hub issues
Website for supporting PA monitors		Incorporated website Q&A and an opportunity to log-in during device training Corresponded regularly with participants to troubleshoot website challenges
Feasibility of using PA-monitoring system		Chose hub locations within churches that were accessible for all participants
Suggestions for improvement		Identified point-person within church community to aid in troubleshooting and correspondence between participants and research team during study period

With ambiguity over project goals as an overall participant concern, we redesigned the written and in-person education component. In training, we explicitly stated project goals verbally and in writing. Additionally, we developed two instructional training videos on device and hub usage that were used during device training and then made publically available after the event for participant reference. We also incorporated more hands-on, in-person training where participants could trial the website, test wristband lights, and upload wristband data.

Due to participant concerns about the hub, we improved our troubleshooting correspondence. We communicated weekly with the device company and the participants to identify and address hub issues, and we incorporated a report sheet next to the hub for streamlined reporting of issues. To address hub availability issues, we provided participants with a schedule of “hub hours” and the option to synchronize their wristbands at any of the participating churches. As per participant recommendation, we identified a point-person within each church community to aid in all troubleshooting and to lead correspondence between participants and the research team during the study period.

## Discussion

### Principal Findings

The objective of this mixed methods pilot study was to engage community members in the evaluation of the feasibility and acceptability of a wrist-worn PA monitor and a CV-health factor tracking website for measuring PA and tracking CV-related health factors prior to implementation in larger groups in similar resource-limited, community-based settings. Both quantitative and qualitative findings revealed that wrist-worn PA monitors can successfully capture and communicate objective PA data (eg, steps, miles, calories, and vigorous activity minutes) on the majority of days and that, contrary to our anticipation, Internet account usage for inputting CV-health related factors was minimal. Strengths and weaknesses of the PA-monitoring system and the user experience were identified and categorized into themes, which improved the PA wristband monitor implementation in our large-scaled study.

This is one of the first studies, to our knowledge, to demonstrate wrist-worn PA monitor systems as a feasible and acceptable means for objectively measuring PA in resource-limited, community-based settings. Our findings suggest that using wrist-worn PA monitors to track PA in resource-limited, community-based populations appears both acceptable and dually beneficial, by providing a feasible way to capture objective PA data and to communicate feedback on PA levels to participants. Additionally, we found that community engagement is critical when integrating new technologies in community-based, resource-limited settings, as it both enhances understanding of the community and the technology environment and aids in identifying strengths and weaknesses of the proposed intervention.

### Wrist-Worn Physical Activity Monitors as Potential Physical Activity Intervention Tool in Community-Based Settings

In a review of PA interventions targeting African American adults, Whitt-Glover et al [21] called for the use of community-based interventions, as “utilizing resources within the community may increase sustainability compared with laboratory-based interventions”. There is evidence suggesting that, despite the practical challenges, technology-driven interventions may succeed in community-based settings [22]. To date, technology-driven PA interventions have incorporated technology largely in two ways: to capture objective measurements (ie, pedometers, accelerometers) [21] or to aid in the delivery of the PA intervention (eg, Internet or text-based interventions, podcasts, mobile apps) [23-26]. Emerging mHealth technologies, specifically wearables, are becoming increasingly available and integrated into interventions that require activity tracking. Few prior studies have tested wrist-worn activity-tracking monitors in a community-based setting [21]. Our findings in a community-based setting, particularly participants’ frequency of PA monitor use and recorded steps, compare favorably to recent studies in noncommunity-based settings testing wearable devices, which have demonstrated that PA interventions incorporating wrist-worn PA monitors are feasible and acceptable within specific populations and can successfully promote and track PA [11,27,28].

Wrist-worn PA monitors track similar quantitative PA data (eg, steps, miles) as the tools used in previous community-based PA studies (eg, pedometers, accelerometers), however, they often have the added benefit of providing comprehensible feedback to the user, a preference highlighted by participants in this study. Feedback from the mHealth wristband was limited in this study; however, the existence of commercial monitors that provide continuous real-time feedback increases the options available to interventionists and could minimize barriers to direct, real-time feedback. The feedback component provides an element of self-monitoring, often described as the “cornerstone” of behavioral interventions [29,30]. Early weight management studies found that more consistent self-monitoring improved weight control and increased the likelihood of participant engagement in the full intervention period [31]. In our study, participants responded positively to a feedback feature, as they also found it aided in self-monitoring, a finding similar among other wrist-worn PA monitor studies [11,27,28]. However, participants preferred a wristband with a more comprehensive and accessible feedback system, particularly one with a more distinct indication of when one enters the “vigorous activity” zone and when one achieves a certain percentage of the daily PA goal. Additionally, participants desired improved accessibility to raw PA data. In our study, participants could access their PA data on their Internet accounts after uploading PA data from their wristband to the central hub at their churches. Participants noted that improved access to feedback would likely increase their self-awareness and self-monitoring, a finding consistent with lifestyle behavior change literature [32-34].

When integrating technology in community-based interventions in resource-limited settings, differential technology access and usage must be acknowledged to reduce potential disparities. This is particularly of concern when developing technology-based interventions that target groups associated with decreased access to the Internet, specifically those of lower socioeconomic status, minority racial group or ethnicity, older age, and poorer health [35-40]. Disparities in wireless broadband adoption are well documented across Washington, D.C. wards. According to a website, wireless broadband adoption in Wards 5, 7, and 8 (66%, 55%, and 58%, respectively) is significantly lower than the remaining D.C. wards, where wireless broadband adoption is greater than 79% [41]. Previous work has demonstrated that utilization of eHealth technologies, predominantly those that relate to health seeking and health tracking, is largely influenced by Internet access and experience in usage [42]. Incorporating a centralized hub did limit participants’ ability to access quantitative PA data in real time; however, it had the added benefit of community-wide accessibility. The hub made uploading and viewing PA data possible for all participants regardless of computer, mobile device, or Internet access. Addressing these well-documented access barriers by incorporating a centralized hub ensures that the PA technology is equally accessible to all participants.

Kumanyika et al [43] highlighted the potential of eHealth and mHealth interventions for addressing obesity in minority youths and adults, calling first for the critical need for evidence to inform the development of eHealth interventions. Findings from this study suggest that an obesity intervention integrating mHealth technology for PA monitoring may be feasible in community-based, resource-limited communities. Advantages of incorporating mHealth technology for PA monitoring in an intervention include real-time data collection and the potential to deliver personalized feedback within the participant’s natural environment. More evidence is needed to determine if incorporating PA-monitoring technology as part of a community-based intervention can improve clinical outcomes in resource-limited settings; however, it does appear to be a feasible and acceptable method to include in future PA studies of similar populations.

### Community-Based Participatory Research as a Useful Approach for Implementing Related Physical Activity Technology in Community-Based Settings

Previous studies have demonstrated that when CBPR principles occur in collaboration with community partners, they serve as sustainable methods of targeting lifestyle factors such as PA and nutrition [44,45]. Using CBPR principles, we implemented and designed this mixed methods pilot study of PA-monitoring technology in collaboration with a CAB, the D.C. CHOC, established in 2012 prior to our large-scale study, the Washington, D.C., CV Health and Needs Assessment. The D.C. CHOC is comprised of a diverse group of community partners and collaborators (six church communities, church leaders, health care providers, leaders from nonprofit organizations, higher education, and local government) to consult on the planning and implementation of the assessment, and the interpretation and dissemination of study findings. Our CAB is a long-term partnership involved in both the CV Health and Needs Assessment and the design and implementation of future community-based behavioral weight loss interventions.

Consistent with CAB responsibilities to represent community members and their input in research activities and “identify key issues for action and strategize next steps” [46], the D.C. CHOC recognized the need to gain insight from and enhance understanding of the targeted community before developing an intervention. Therefore, they recommended a focus group and pilot study to test the acceptability and feasibility of the new PA technologies to be used in the large-scaled study. This step would not have been considered without a CBPR framework, thus demonstrating that novel ideas originate when research questions stem from community partnerships in the local context.

Our mixed methods pilot study revealed that community engagement is a critical component of community-based intervention development, as it allows for testing specific elements of an intervention that otherwise would be challenging without community support. Integrating a technology-based intervention in resource-limited communities requires researchers to first understand the community’s technology environment and barriers to usage. If adequate knowledge is not obtained during the preliminary intervention development stages, interventions run the risk of being initiated prematurely and out of context. Engaging community members during the pilot study and focus group resulted in community-specific suggestions and improvements that we implemented in our large-scaled CV Health and Needs Assessment and plan to implement in a future behavioral weight-loss intervention. By engaging community members, we tailored the PA-monitoring technology to the specific community context. Our study showed that community insight within the CBPR framework allows for a research team to anticipate the community’s future technological needs for an intervention.

CBPR methods also allowed us to tailor the implementation of our PA-monitoring system to the unique needs of the community members. Previous work suggests that behavioral weight loss interventions in African American, church-based settings can be effective if specific PA tools that promote weight loss are provided [47]. Before integrating such tools into an intervention, it is necessary to determine if the tools are feasible and acceptable for personal PA-monitoring in community-based populations. By engaging community members, we were able to gain insight into the strengths and weaknesses of our proposed PA-monitoring system. In particular, the focus group feedback enabled us to enhance our training and larger implementation (eg, identified hub point-persons, expanded hub hours), to address concerns (eg, wristband irritation, wristband falling off), and to clarify vague instructions (eg, vigorous activity definition, colored-light system). Our study demonstrates that incorporating CBPR principles is a necessary step during intervention development, especially when introducing technology-based PA-monitoring systems to resource-limited communities. This is particularly relevant for larger-scaled studies stemming from this pilot study, as CBPR research shows that incorporating community members’ feedback enhances the relevance of a study, may improve sustainability of the proposed intervention, and may potentially improve the retention of study participants [44,45,48].

### mHealth Technology Is Feasible for Physical Activity Interventions in Resource-Limited Communities

Our study has shown that, though certain challenges do exist, a wrist-worn PA monitor could be an mHealth tool used to monitor and facilitate PA for weight management in resource-limited, community-based settings. Recent work has demonstrated the effectiveness of eHealth and mHealth interventions for weight management; however, little is known about the success of mHealth PA interventions in community-based populations, particularly those that are resource-limited [45]. It is known, however, that the once wide “digital divide” (ie, the gap in computer and Internet access across racial/ethnic minorities) is narrowing due to the expansion of mobile computing options [49]. As mobile phone usage increases, so too does the use of mHealth technology for health tracking [39,50].

Personal mobile devices offer a platform for health tracking and an opportunity to minimize costs and burden for the individual. Preliminary evaluation studies show that wearable devices (in addition to mobile phone step-tracking apps) accurately measure step counts when compared to manually and accelerometer counted steps, a finding that may alleviate reservations regarding mobile phone apps and wearables for PA monitoring [12]. In our study, participant PA data were successfully captured by the PA-monitoring system on the majority of days and monitored by the participant. For syncing and self-monitoring PA, participants with mobile phones preferred alternative options; such as a mobile phone app. Future studies should expand on this work by providing convenient and accessible options for syncing PA data in the community and on personal devices, as it may facilitate success and sustainability of wearable PA tracking in community-based settings.

### Strengths and Limitations

Strengths of this study include the community-based, resource-limited nature of our setting, the novel use of technology with a community-based population, the incorporation of CBPR strategies, and the combination of qualitative and quantitative data gathered from the pilot testing. However, limitations of this study must be acknowledged. The study was short, limiting testing of participant adherence, engagement, retention, and attrition. Future work would benefit from a longer study period to gauge these factors. Additionally, generalizability may be a concern, as the sample size was small, and participants were recruited from the same African American, faith-based communities that would be targeted in a follow-up observational study. Additionally, while all subjects were African American, it is important to note that most had high levels of education, so study findings also would not be generalizable to a population with low levels of education. Future work would benefit from extension of this study to a larger and more diverse sample. Preferences and suggestions made in the focus group may not be representative of the target population, as responses were gathered in a group setting, with no opportunity to provide confidential responses.

While our study captured feasibility and acceptability of a potential technology-based tool for objectively capturing PA, our study did not intend to test the effectiveness of the PA-monitoring technology in modifying behavior. Future research should also include effectiveness in the study outcomes. While there has been progress incorporating technology in community-based PA interventions, more work, particularly around mobile device access and usage, digital literacy, and locations of publically accessible wireless Internet connections, is needed to improve our understanding of potential technology-based interventions in resource-limited communities. While the proposed PA-monitoring system used in this study allowed for assessing preliminary feasibility and acceptability, it does present limitations in the context of dissemination and implementation across diverse settings. As popular, commercially available PA-monitoring devices such as the Fitbit continue to advance, more accessible and affordable options will likely emerge that may support widespread implementation across diverse, low-resource communities more adequately than this study’s proposed system.

### Conclusions

A wrist-worn PA-monitoring system appears to be a feasible and acceptable technology for potential use in larger-scaled studies in community-based, resource-limited settings. CBPR methods, particularly CABs and focus groups, aid in early identification of issues, suggestions, and preferences as they relate to technology implementation in community-based, resource-limited settings. Additional work is needed to evaluate the effectiveness of and engagement with PA-monitoring systems in this setting. While a multifaceted behavioral intervention combining behavior change elements, dietary therapy, and PA is likely needed for weight loss management, this study provides evidence to support use of PA-monitoring technologies as part of a PA intervention in larger scaled studies in resource-limited, community-based settings in Washington, D.C. Of equal importance, this pilot demonstrates that in an era of limited funding and widening health disparities, we can ill afford not to engage the community leaders and community members in partnerships where clinicians, researchers, and community members can leverage the strength of their collaboration to design and implement health behavior studies that are both feasible and acceptable to the communities they target.

## Appendix

Multimedia Appendix 1 Focus group Moderator's Guide- Cardiovascular Health and Needs Assessment Qualitative Study, 2014.

Multimedia Appendix 2 Criteria for judging scientific rigor in qualitative research.

## Abbreviations

app: applications
CAB: community advisory board
CBPR: community-based participatory research
CV: cardiovascular
CVD: cardiovascular disease
D.C. CHOC: D.C. Cardiovascular Health and Obesity Collaborative
eHealth: electronic health
mHealth: mobile health
NIH: National Institutes of Health
PA: physical activity
PMI: Precision Medicine Initiative
