# Supplementary material for: Community Engagement to Optimize the Use of Web-Based and Wearable Technology in a Cardiovascular Health and Needs Assessment Study: A Mixed Methods Approach
Source: JMIR Mhealth Uhealth. 2016 Apr 25;4(2):e38. doi: 10.2196/mhealth.4489 (PMC4861844; doi:10.2196/mhealth.4489)
Supplement: Multimedia Appendix 1 [file mhealth_v4i2e38_app1.pdf]

Multimedia Appendix 1

Focus group Moderator's Guide- Cardiovascular Health and Needs Assessment Qualitative Study, 2014

FOCUS GROUP MODERATOR'S GUIDE FOR CARDIOVASCULAR HEALTH AND NEEDS ASSESSMENT IN WASHINGTON DC

Drafted by Dr. Gwenyth Wallen, R.N., PhD

SESSION II - FOCUS GROUP ON DEVICES

II. Group Discussion/ Interviewing Session

- We will welcome the participants to Session II of the focus group series and thank them for their cooperation.

- We will ask the participants for their diary on their use and experiences with the devices for measuring physical activity and dietary intake.

- We will explain to the participants the purpose of this session regarding their experience with the devices for measuring physical activity and dietary intake. There would be another set of preliminary questions to begin the session, but the facilitators will ask other supporting questions to probe for participant feedback in feasibility and comfort with devices for measuring physical activity and dietary intake.

NOTE: During the group session, we will be recording during this period to ensure we collect all the feedback and information exchange from the session. This session will last up to 1.5 hours.

III. Key Questions

A. Physical Activity Device Follow-up

Remember: Please continue to probe after each question to get participant's perspective on the use of the device.

1. ***Did everyone follow the instructions provided to you on the devices in Session I?***

2. ***Did anyone have problems or issues using the devices in the last two weeks?***
   a. ***If so, could you tell us of the problems?***

3. ***Did anyone have difficulty or have issues with collecting the data from the devices?***

4. ***Did anyone have difficulty or problems with downloading the data from the devices to the server?***

5. ***Were any of you able to access and review your data results on the internet, (list website)?***
   a. ***If you were not able to access the site or retrieve your data results, what were the reasons that hindered you from retrieving the data?***
   b. ***If any problems occur with the internet site, can you tell us of those issues and examples of the types of issues?***

6. ***Did you have any skin irritation, rash, pain or physical issues while wearing the device for monitoring physical activity?***
   a. ***If so, can you tell us of those issues?***

7. ***Did wearing the physical activity device encourage you to increase your own physical activity and exercise more often?***

B. *Digital Camera Follow-up*

Remember: Please continue to probe after each question to get participant's perspective on the use of the device.

8. ***For persons using the digital camera, did you follow the instructions provided during the training in Session I?***

9. ***Did any problems or issues occur using the digital camera in the last two weeks?***
   a. ***If so, could you tell us of the problems?***

10. ***Did anyone have difficulty or problems with downloading the data from the camera to the server?***

11. ***Were any of you able to access and review your data results from the camera on the internet, (list website)?***
    a. ***If you were not able to access the site or retrieve your data results, what were the reasons that hinder you not retrieving the data?***
    b. ***If any problems occur with the internet site, can you tell us of those issues and examples of the types of issues?***

C. *Wrap-up*

- ***Please ask the participant for recommendations or suggestions for feasibility and comfort in using the physical activity devices and digital camera***

Remember: Please continue to probe after each question to get participant's perspective on the use of the devices.

12. ***What are your overall thoughts and feedback regarding these devices?***

13. ***Also, do you feel that these devices would be an asset for people who want to lose weight or improve their overall health?***

14. ***Do you think people in your community would use these devices?***

15. ***Overall, do you feel that persons in your community would like to have and use these devices?***

16. ***Do you think it would be a waste of time to offer these devices?***
    a. ***If so, why do you believe this is the case?***

17. ***Do you have any suggestions on other things you would like to see or use if these devices were offered to you and others in your community?***

18. ***What do you see as potential barriers for people using these devices in your community?***

Remember: Please continue to probe after each question to get participant's perspective on the use of the devices.

IV. Closing

- After the final question, the moderator would again thank the participants for returning to session II and taking part of this focus group project.

- The moderator and principal investigator will express their appreciation for comments and information exchange from the participants of the focus group.

- Moderator will inform the participants that their feedback will be a part of a process of designing the best way to administer the study questionnaire to participants

- The moderator will remind the participants about confidentiality in discussion of this study with others.

- Participants will receive remuneration for their participation in this focus group

THANK YOU AGAIN AND HAVE A GOOD DAY/EVENING
