# Supplementary material for: Community Engagement to Optimize the Use of Web-Based and Wearable Technology in a Cardiovascular Health and Needs Assessment Study: A Mixed Methods Approach
Source: JMIR Mhealth Uhealth. 2016 Apr 25;4(2):e38. doi: 10.2196/mhealth.4489 (PMC4861844; doi:10.2196/mhealth.4489)
Supplement: Multimedia Appendix 2 [file mhealth_v4i2e38_app2.pdf]

Multimedia Appendix 2

Criteria for judging scientific rigor in qualitative research

**Criteria**	**Criteria characteristics**	**Example methods for this study**
Credibility	"Truth of findings as judged by participants and others within the discipline. For instance, you may find the researcher returning...to share interpretation of findings and query accuracy from the perspective of the persons living the experience."	Validation of themes by an intramural mixed-methods expert
Auditability	"Accountability as judged by the adequacy of information leading the researcher from the research question and raw data through various steps of analysis to the interpretation of findings. For instance, you should be able to follow the reasoning of the researcher step-by-step through explicit examples of data, interpretations, and syntheses."	When publishing, showing examples of data alongside its corresponding themes; detailed description of the iterative process of coding and multiple coders coming to consensus (how many coders, how many sessions, % initial disagreement, etc)
Fittingness	"Faithfulness to everyday reality of the participants, described in enough detail so that others in the discipline can evaluate importance for their own practice, research, and theory development. For instance, you will know enough about the human experience being reported that you can decide whether it 'rings true' and is useful for guiding your practice."	Collecting enough data so that saturation is reached, allowing for inferences to be drawn with direct implications for practice, research, and/or theory development

Quoted and adapted from Barroso, J. (2010). Qualitative approaches to research. In G. LoBiondo-Wood and Haber, J. (Eds). *Nursing Research: Methods and Critical Appraisal for Evidence-Based Practice.* Elsevier: St. Louis, MO, USA.
